# The miRNA Expression Profile in Acute Myocardial Infarct Using Sheep Model with Left Ventricular Assist Device Unloading

**DOI:** 10.1155/2017/4352450

**Published:** 2017-09-11

**Authors:** Xiaoqian Yan, Yu Gan, Haibo Chen, Guangmao Liu, Shengshou Hu, Jianye Zhou

**Affiliations:** ^1^State Key Laboratory of Cardiovascular Disease, Fu Wai Hospital, National Center for Cardiovascular Diseases, Chinese Academy of Medical Sciences and Peking Union Medical College, Beijing 100037, China; ^2^Dongguan People's Hospital, Dongguan, Guangdong 523059, China

## Abstract

This study attempted to establish miRNA expression profiles in acute myocardial infarct (AMI) sheep model with left ventricular assist device (LVAD) unloading. AMI was established in sheep model and FW-II type axial flow pump was implanted to maintain continuous unloading for 3 days. The cardiomyocyte survival, inflammatory cell infiltration, and myocardial fibrosis were detected by tissue staining, and cardiomyocyte apoptosis was detected by TUNEL assay. High throughput sequencing technique was used to detect miRNA expression in cardiomyocytes and to establish miRNA expression profile. The Gene Ontology (GO) and Kyoto Encyclopedia of Genes and Genomes (KEGG) analyses were established. miRNA sequencing results identified 152 known mature miRNAs and 1582 new mature miRNAs. The unloading and control groups differentially expressed genes, of which RT-PCR verified oar-miR-19b and oar-miR-26a. The GO and KEGG pathway annotation and enrichment established that the regulating functions and signaling pathways of these miRNAs were closely related to cardiovascular diseases (CVD). In this study, LVAD effectively reduced the cell death degree of cardiomyocyte in MI. The established miRNA expression profiles of AMI and LVAD intervention in this study suggest that the expression profile could be used to explore the unknown miRNA and the regulatory mechanisms involved in AMI.

## 1. Introduction

Since cardiomyocytes lack regenerative ability, ischemia and hypoxia can cause myocardial cell injury leading to cardiomyocyte loss and fibrous tissue hyperplasia, causing ventricular remodeling and progressive heart failure (HF). The LVAD is proven to be an effective therapy for HF and can be used for mechanical circulatory support through the unloading way to bring a beneficial effect on the left ventricular failure [1]. Unloading can reverse myocardial compensatory response and stimulate the changes on cellular and molecular levels in the failing heart, leading to improved myocardial function and eventually resulting in the structural and functional “reverse remodeling” of myocardial tissue [2, 3].

For the treatment of AMI, researchers found that it was of great significance to apply LVAD therapy in time before the cardiogenic shock leads to irreversible organ failure [4]. Sun et al. [5] used pigs as models and found that the use of LVAD at the early stage of AMI could limit left ventricular remodeling, protect myocardial systolic function at the late stage of AMI, and improve the prognosis. At present, the prognosis and mechanism of LVAD at the early stage of AMI remain to be elucidated.

MicroRNA (miRNA) is an endogenous noncoding single-stranded small molecule RNA composed of 21–23 base pair nucleotides and can silence the target gene on the posttranscriptional level to achieve fine regulation of biological genes [6]. Previous studies have shown that miRNAs were involved in myocardial hypertrophy, myocardial ischemia, arrhythmia, and other pathophysiological processes [7–11] and were a kind of important endogenous regulatory factors. At present, studies on the function and mechanism of miRNAs in myocardial ischemia are still at the initial stage, especially those on changes in miRNAs during the early stage of myocardial ischemia. Currently, there are no reports on analysis of miRNA in myocardial cells after LVAD intervention at the early stage of AMI or studies on its related signal pathways.

The present study established a sheep model of AMI and used the self-developed FW-II axial flow ventricular assist pump for early intervention. The miRNAs of cardiomyocytes in the normal myocardial tissue, infarcted myocardial tissue, and infarction border zone were sequenced, respectively, to obtain miRNA expression profiles of the three tissues, which provided basic data for further analysis on the mechanism of myocardial remodeling and reverse remodeling and searching for important signaling pathways and key molecular targets.

## 2. Methods and Methods

### 2.1. MI Modeling and Unloading Detection

Twelve adult male Small-Tail Han sheep, each weighing 54.2 ± 8 kg, were randomly divided into unloading group (MI + VAD; *n* = 5) and control group (MI; *n* = 7), respectively. The animals were given heparin (3 m/kg) intravenously and the activated blood clotting time (ACT) was maintained above 400 s after propofol-induced anesthesia was successfully performed. Endotracheal intubation was performed and isoflurane was inhaled for general anesthesia maintenance. The fourth left intercostal space was selected for operative incision. Lidocaine was sprayed on the surface of the heart. The root segment of the obtuse marginal branch was ligated by 5-0 prolene line. The color of obtuse marginal branch distributed cardiac tissue was observed purpling, which was regarded as the successful MI modeling.

For the animals in the unloading group, the input of FW-II type axial flow circulation auxiliary pump was inserted into the left ventricle through the apex of heart before MI modeling. Then the output was end-to-side anastomosed to descending aorta, and exhaustion and the reliability of connection were confirmed. While the animal model of MI was successfully performed, the FW-II type axial flow pump was turned on, and the flow rate was set closely to the preoperative cardiac output. The Swan-Ganz catheter was placed in the heart for cardiac output and pulmonary artery wedge pressure observation, and the success of unloading was confirmed. The anastomotic bleeding was confirmed not to appear. The speed of blood pump was 8000 rpm, and the flow maintained at around 2.2 L/min. Negative pressure drainage was placed in left thoracic cavity. The thoracic cavity was closed in the routine process.

The ECG detection was performed both before surgery and after modeling. After surviving for 72 hours, animals were sacrificed with potassium chloride during which the auxiliary pump was stopped. The animal heart was removed completely, and each part of MI, Infarct Border Zone, and normal myocardial tissue was taken for miRNA extraction. The remaining tissues were fixed in 10% formalin solution for reserve.

All animal experiments were approved by the animal ethics committee of Fuwai Hospital, Chinese Academy of Medical Sciences, and strictly abide by the regulations on animal protection of Fuwai Hospital.

### 2.2. Pathological Staining and Apoptosis Detection of Myocardial Cell

The heart tissues from Infarct Zone, Infarct Border Zone, and normal zone were taken in unloading group and control group, respectively. The tissues were then fixed, embedded, sectioned and stained using Hematoxylin-Eosin (HE), Masson, and Sirius red. Four paraffin blocks were randomly selected by digital methods from Infarct Zone and Infarct Border Zone in each group, respectively. Then TUNEL cell apoptosis assay was performed. Three photos were taken from each paraffin block and the proportion of apoptosis was calculated.

### 2.3. miRNA Sequencing

For RNA extraction, 30–120 mg of heart tissue samples was used. Tissue was ground and resuspended in QIAzol Lysis Reagent (Qiagen). Total RNA was extracted by using phenol-chloroform extraction methods according to the manufacturer's instructions of miRNeasy kit (Qiagen). The RNA 6000 Pico Kit (Agilent Technologies 2100 Bioanalyzer) was used for quality control of total RNAs. Small RNA libraries were constructed according to the manufacturer's instructions of the TruSeq Small RNA kit (Illumina). Sequencing was performed by using Illumina's Hiseq 2500. Raw sequences of 50 bp length were produced and demultiplexed using the Illumina pipeline CASAVA v1.8. Adapter sequences should be initially trimmed from primary reads by using FASTX-toolkit 0.0.14 [12]. The high-quality sequence was maintained with the length of 18–30 nt after trimming again. High-quality sequences clean reads were obtained after data processing, which is small RNA. Then statistics of small RNA length were performed. The sequences alignment were performed between the obtained sequences and the sequences in miRBase v21 [13] database, Rfam 12.0 [14] database, SILVA 115 [15] database, and Repbase 20.04 [16] database, respectively. Different small RNAs were classified and at last the unclassified clean reads were used for new miRNA prediction. The* Ovis aries* (sheep) reference genome was selected as the reference genome in this sequencing. The link of* Ovis aries* (sheep) reference genome was as follows:

ftp://ftp.ensembl.org/pub/release-79/fasta/ovis_aries/dna/Ovis_aries.Oar_v3.1.dna_sm.toplevel.fa.gz. The link of annotation document of* Ovis aries* (sheep) reference genome was as follows: ftp://ftp.ensembl.org/pub/release-79/gtf/ovis_aries/Ovis_aries.Oar_v3.1.79.gtf.gz. The reads which were matched for the reference genome were used for miRNA analysis by miRDeep2 [17] software. That is identifying the known miRNA, predicting new miRNA, and analyzing the conservatism of new miRNA. The correlation among samples was assessed by using Pearson's correlation coefficient. The differential expression analysis of miRNA was performed between grouping samples and the upregulated and downregulated miRNAs were obtained. The differential expression analysis of miRNA expression level was performed by using the edgeR [18] package in R language. The differentially expressed miRNA between samples was screened. The significant *P* value and fold change (FC) were used as the criterion for judgement of differential expression: (a) *P* value ≤ 0.05 and log⁡FC ≥ 1 (FC ≥ 2) were considered as upregulated miRNA; (b) *P* value ≤ 0.05 and log⁡FC ≤ −1 (FC ≤ 0.5) were considered as downregulated miRNA.

### 2.4. Validation by Quantitative Real-Time PCR

The expression analyses of specific miRNAs were confirmed by using quantitative real-time (Q-RT) PCR. The cDNA was obtained from 1 *μ*g of total RNA by using the 1st Strand cDNA Synthesis Kit (Takara). Q-RT-PCR was performed by using SYBR FAST qPCR Kit with Master Mix (2x) and Universal (KAPA Biosystems).

### 2.5. GO and KEGG Analysis

Target gene prediction of the differentially expressed miRNA was performed by using target gene prediction software PITA [19] and miRanda [20]. All genes were mapped to each entry of GO (Gene Ontology) database (http://www.geneontology.org/) in the GO significant enrichment analysis. The number of genes in each GO Term was calculated, and then by comparing with the set of all genes in the whole genome background, the hypergeometric test was performed to find the significantly enriched GO Term from the target genes of differentially expressed miRNA. KEGG [21] is a main public database in pathway analysis, KEGG Pathway was used as a unit of the Pathway significant enrichment analysis. The hypergeometric test was performed to find significantly enriched pathway from all genes in the whole genome background. The main biochemical pathways and signal transduction pathways could be identified by pathway enrichment analysis.

### 2.6. Statistical Analysis

Apoptosis ratio and Q-RT data were represented as mean ± SE. Student's *t*-test was used for comparison between the two groups. Pearson's correlation coefficient (*R*) was calculated for correlation analysis. *P* value ≤ 0.05 indicated that the difference was statistically significant.

## 3. Results

### 3.1. Myocardial Infarct Model Making and Unloading

Of the 5 cases in the unloading group, 3 cases survived, 1 case died of ventricular fibrillation, and 1 case died of postoperative cardiac rupture. Of the 7 cases in the control group, 3 cases survived and 4 cases died of ventricular fibrillation.

**Figure 1:**
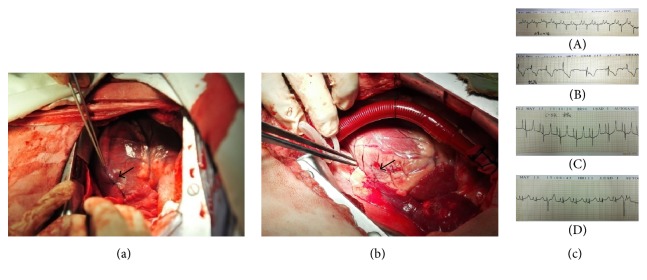
Myocardial infarct model making and ECG detecting. (a) Obtuse marginal branch of the control group ligation parts; (b) unloading group implanted with LVAD and the same as the control group ligation parts. In (c), (A) is preoperative ECG of unloading group; (B) is ECG of unloading group after coronary artery ligation; (C) is preoperative ECG of control group; (D) is ECG of control group after coronary artery ligation.

The results of MI modeling are shown in Figures 1(a) and 1(b). The color of obtuse marginal branch distributed cardiac tissue was observed purpling (indicated by the arrow in Figures 1(a) and 1(b)). Abnormal heart beat and increased heart rate were observed, and myocardial ischemia was detected by electrocardiogram (Figure 1(c)).

The detection results of the Swan-Ganz catheter (Table 1) showed that the cardiac output in myocardial infarct model significantly decreased (*P* < 0.05) compared with the preoperative cardiac output. Both ventricular volume loading and pulmonary artery wedge pressure significantly increased (*P* < 0.05) after myocardial infarct modeling. Cardiac output was significantly increased (*P* < 0.05) while pulmonary wedge pressure significantly decreased (*P* < 0.05) after the FW-II type axial flow pump was started. The volume loading of the left ventricle was decreased after the FW-II type axial flow pump was started.

### 3.2. Sample Examinations

#### 3.2.1. The Results of Histological Examination

**Figure 2:**
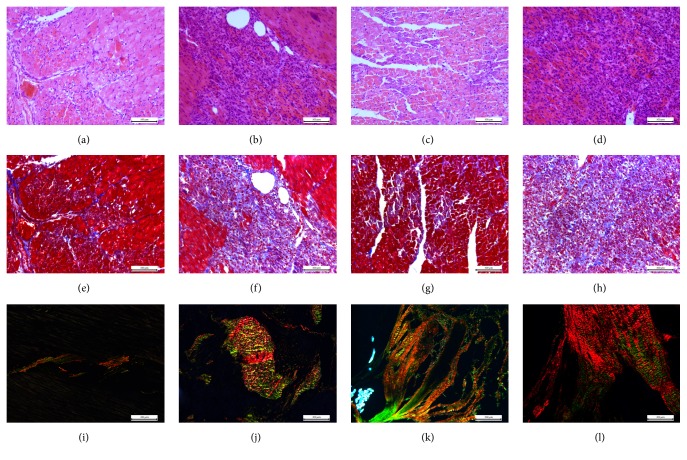
HE staining (a–d), Masson staining (e–h), and Sirius red staining (i–l) of different positions of myocardial tissue of unloading group and control group. (a), (e), and (i) are Infarct Border Zone of unloading group; (b), (f), and (j) are Infarct Border Zone of control group; (c), (g) and (k) are Infarct Zone of unloading group; (d), (h), and (l) are Infarct Zone of control group.

The results of HE staining were shown in Figures 2(a)–2(d); pathological staining showed the destruction of a large amount of cardiac myocytes and inflammatory cells exudation in the control group. In the unloading group, a weakened destruction of cardiac myocytes was observed, while a significant reduction in inflammatory cells infiltration was also seen. Cardiomyocyte necrosis was significantly reduced.

In the Masson staining, the red region represented cardiac myocytes, while the blue region represented collagen fibers (Figures 2(e)–2(h)). In the unloading group, the areas of collagen decreased and a lot of cardiac myocytes were reserved in both of the Infarct Broad Zone and Infarct Zone.

The results of Sirius red staining were shown in Figures 2(i)–2(l). The degree of collagenization was significantly alleviated in the unloading group. Type III collagen was the main type of collagen in Infarct Zone of the unloading group. Type I collagen was the main type of collagen in the Infarct Zone of the control group. Type I collagen showed complete loss of elasticity compared with type III collagen.

#### 3.2.2. Apoptosis of Cardiac Myocytes

**Figure 3:**
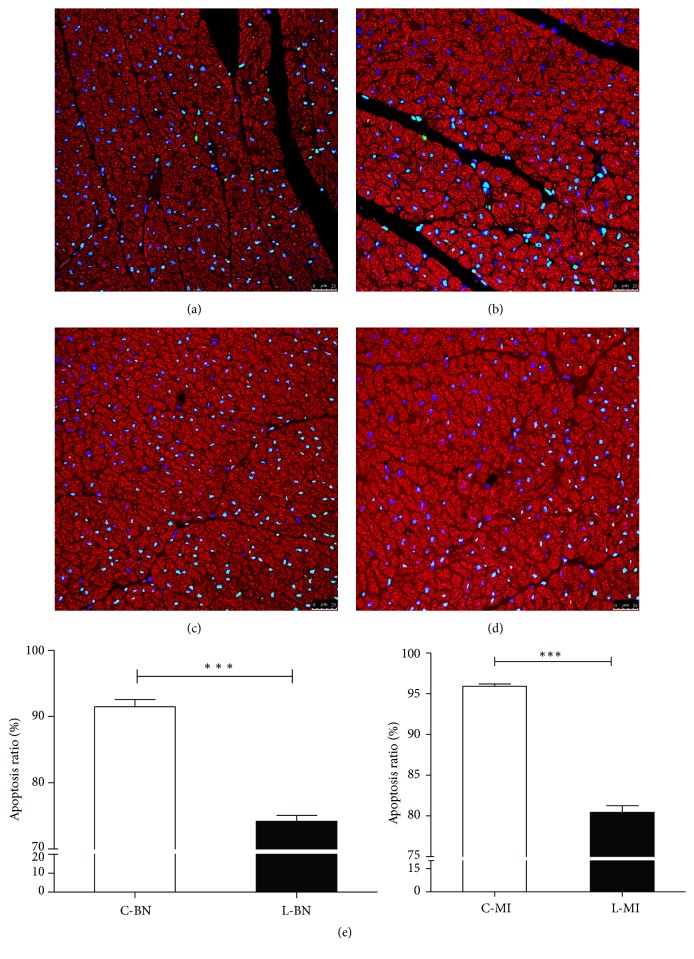
The contrast of apoptosis ratio of Infarct Zone (a, c) and Infarct Border Zone (b, d), respectively, in the control group (a, b) and unloading group (c, d), *P* < 0.001.

The normal nuclei showed blue while the apoptotic nuclei showed yellow-green after being excited by fluorescence. In the unloading group, the apoptosis of cardiac myocytes was significantly weakened compared with control group (see Figure 3). The proportion of apoptosis was significantly decreased (*P* < 0.001) (see Figure 3(e)). These results indicated that the animal model of myocardial infarct was successfully performed, and the unloading effect was accurate.

### 3.3. miRNA Sequencing and Analysis

#### 3.3.1. miRNA Sequencing

**Figure 4:**
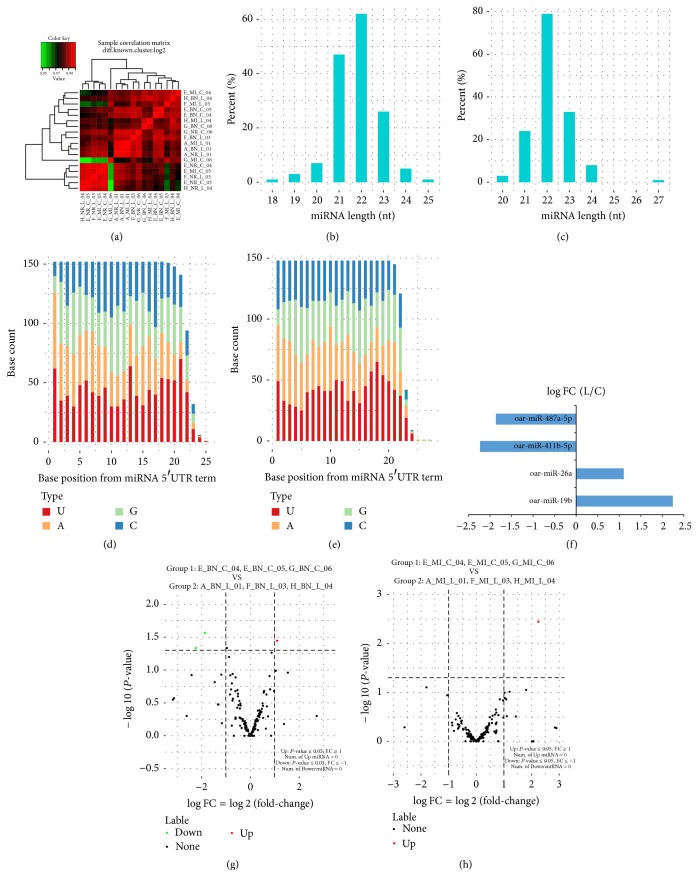
The result of miRNA sequencing. (a) The heatmap of the relationship (according to the known miRNA expression); (b, c, d, and e) the length and base distribution map of known (b, d) and new (c, e) miRNA; (f) differentially expressed miRNA in Infarct Zone and Infarct Border Zone of unloading group and control group; (g) miRNA Volcano Plot (the contrast of Infarct Border Zone in the unloading group and control group); (h) miRNA Volcano Plot (the contrast of Infarct Zone in the unloading group and control group).

Pearson's correlation coefficient between samples was calculated according to the normalized miRNA expression level in each sample. The heatmap of the relationship is shown in Figure 4(a), which shows a high correlation of samples, good repeatability of animal experiments, and sampling. The results of miRNA microarray from different parts of myocardial tissue in unloading group and control group are shown in Table 2. The length and base distribution map of miRNA are shown in Figures 4(b), 4(c), 4(d), and 4(e). The differential expression analysis of miRNA was performed between grouping samples, and the upregulated and downregulated miRNAs were obtained.

The differentially expressed miRNA between samples were screened. The significant *P* value and fold change (FC) were used as the criterion for judgement of differential expression.

Compared with control group, the differentially expressed miRNA in Infarct Zone was oar-miR-19b in the unloading group, while there were three differentially expressed miRNA in Infarct Border Zone (Figures 4(f), 4(g), and 4(h)) including downregulated oar-miR411b-5p and oar-miR-487a-5p and upregulated oar-miR-26a.

#### 3.3.2. Real-Time Fluorescent Quantitative PCR for Differentially Expressed miRNA Verification

**Figure 5:**
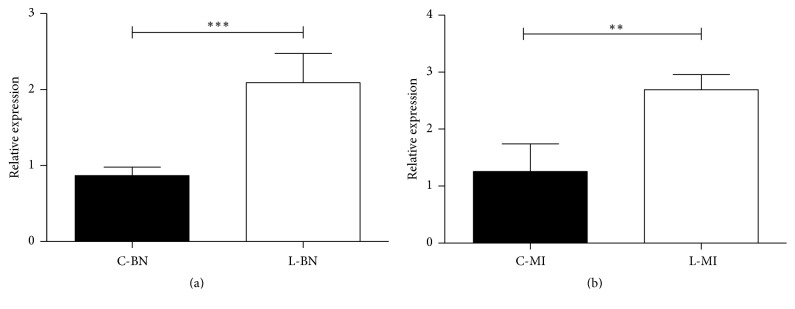
Verification (qPCR) of the differentially expressed miRNAs including (a) oar-miR-26a and (b) oar-miR-19b (*∗∗* means  *P* < 0.05; *∗∗∗* means *P* < 0.01).

The RT-PCR was used for verification of the differentially expressed miRNAs including oar-miR-19b, oar-miR-26a, oar-miR411b-5p, and oar-miR-487a-5p, which were screened by microarray. The results are shown in Figure 5. The difference in oar-miR-19b (*P* < 0.05) and oar-miR-26a (*P* < 0.01) expression between unloading group and control group was significant. The results were consistent with the microarray, while the expression of oar-miR411b-5p and oar-miR-487a-5p showed no significant difference.

#### 3.3.3. GO and KEGG Analysis of Validated Differentially Expressed miRNA

**Figure 6:**
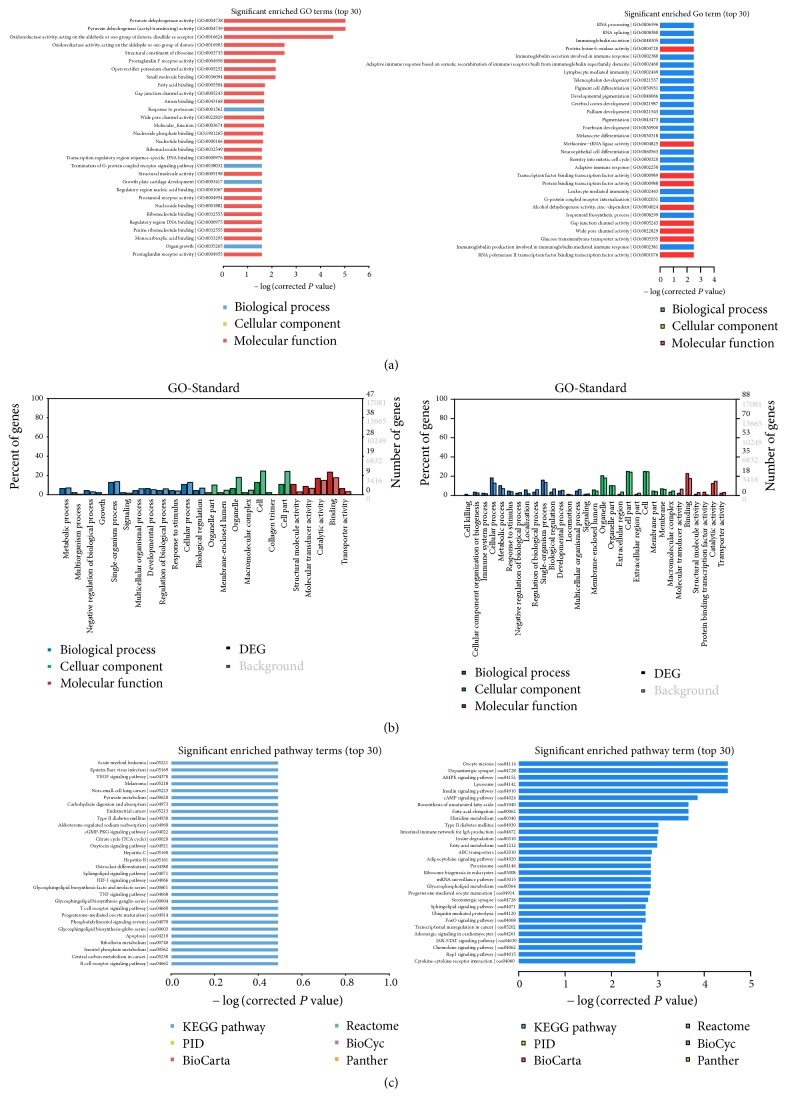
GO and KEGG analysis of differentially expressed miRNA. (a) Significantly enriched GO terms (TOP30) of oar-miR-19b (left) and oar-miR-26a (right); (b) GO-Standard of oar-miR-19b (left) and oar-miR-26a (right); (c) significantly enriched pathway terms (TOP30) of oar-miR-19b (left) and oar-miR-26a (right).

The genes were classified according to Cellular Component, Molecular Function, and Biological Process by using GO analysis. KEGG is a main public database on pathway analysis. Pathway enrichment analysis identified the main biochemical pathways and signal transduction pathways. The GO and KEGG pathway annotations and enrichment were performed in two differentially expressed miRNAs (oar-miR-19b and oar-miR-26a) as shown in Figure 6.

## 4. Discussion

The HF is associated with remodeling, which includes the reverse changes of myocardial cells, structures, and functions [22]. The LVADs perform mechanical unloading in advanced HF and could improve the symptoms and end-organ perfusion [23]. It is also believed to have the ability to stimulate cellular and molecular responses [2, 24], which can reverse maladaptive myocardial remodeling [3]. However, the mechanism of applying for mechanical support after AMI is still unclear [25].

In our experiments, FW-II axial flow pump was applied to assist the circulation of hearts in sheep model with AMI. Results showed that the use of LVAD at the early stage of MI had obvious unloading effects on infracted myocardial tissue. Evidence from the histological results showed reduced apoptosis of cardiomyocytes, significantly reduced infiltration of inflammatory cells, and retarded development of myocardial fibrosis. The results of unloading by using FW-II type axial flow pump were similar to the classic unloading effect described by Jung et al. [26]. The effect of unloading was exact. According to the current opinion, all those changes were supposed to be regulated originally by miRNAs.

miRNAs are important regulators of the remodeling process. A comparative miRNA profiling in the sheep hearts with AMI with/without LVAD assistance could help to understand the underlying molecular mechanisms.

In this study, 152 known mature miRNAs and 1582 new mature miRNAs were found, and among them 815 were conservative. The involved functions and signaling pathways were aggregated and displayed by bioinformatics, which laid a solid foundation for the subsequent exploration on the mechanism of LVAD on cardiac remodeling and reverse remodeling after AMI. For example, the differentially expressed miRNA in the infarcted area was oar-miR-19b; the differentially expressed miRNAs in the infarct boundary area included downregulated oar-miR411b-5p, oar-miR-487a-5p, and upregulated oar-miR-26a. The results of oar-miR-19b and oar-miR-26a were verified by q-PCR. Through the analysis of GO and KEGG, oar-miR-19b was related to the functions of open rectifier potassium channel activity, gap junction channel activity, anion binding and the signaling pathways of VEGF signaling pathway, and apoptosis signaling pathway. The oar-miR-26a was related to the functions of reentry into the mitotic cell cycle, leukocyte mediated immunity, gap junction channel activity, and the signaling pathways of Lysosome, Jak-STAT signaling pathway, and adrenergic signaling in cardiomyocytes. Both the functions and pathways were closely related to CVDs and might play a role in cardiac remodeling and reverse remodeling. For example, activation of VEGF signaling pathway can promote angiogenesis. Activation of apoptotic signaling pathway can regulate the process of cardiomyocyte apoptosis. JAK-STAT pathway activation can prevent the occurrence of myocardial apoptosis after infarction. In addition, both miR-19b and miR-26a had a human origin and could be further studied. Next we will do miR-19b and miR-26a function validation further. In addition, we found 1582 new mature miRNAs, which also helped us to discover unknown miRNAs, even species-specific new miRNAs.

This study has found not only the known differential expressed miRNAs but also many unknown differentially expressed miRNAs. Comparing the unloading group with the control group (see Supplementary Material available online at https://doi.org/10.1155/2017/4352450), there were 16 new differentially expressed miRNAs in the infarcted area, 92 new differentially expressed miRNAs in the infarcted boundary area, and 36 new differentially expressed miRNAs in the normal myocardial region. Through GO analysis and KEGG analysis, these functions and signaling pathways were found to be closely related to CVD. Therefore, the established miRNA expression profile could help not only to explore the known molecular pathways but also to discover new mechanisms and find the response differences between human and sheep species.

## 5. Conclusion

In this study, LVAD effectively reduced the cell death degree of cardiomyocyte in AMI after unloading three days. The established and validated miRNA expression profiles of AMI and LVAD intervention in this study suggest that the expression profile could be used to explore the unknown miRNA, including their function in AMI and LVAD unloading, and investigate the regulatory mechanisms involved in AMI.

## Supplementary Material

Table S1: Differentially expressed and new miRNA of Infarct Zone in unloading group.
Table S2: Differentially expressed and new miRNA of Infarct Border Zone in unloading group.
Table S3: Differentially expressed and new miRNA of Normal Zone in unloading group.
Figure S1: Significant enriched GO terms (TOP30) of differentially expressed and new miRNA of Infarct Zone in unloading group.
Figure S2: Significant enriched GO terms (TOP30) of differentially expressed and new miRNA of Infarct Border Zone in unloading group.
Figure S3: Significant enriched GO terms (TOP30) of differentially expressed and new miRNA of Normal Zone in unloading group.
Figure S4: GO-Standard of differentially expressed and new miRNA of Infarct Zone in unloading group.
Figure S5: GO-Standard of differentially expressed and new miRNA of Infarct Border Zone in unloading group.
Figure S6: GO-Standard of differentially expressed and new miRNA of Normal Zone in unloading group.
Figure S7: Significant enriched Pathway terms (TOP30) of differentially expressed and new miRNA of Infarct Zone in unloading group.
Figure S8: Significant enriched Pathway terms (TOP30) of differentially expressed and new miRNA of Infarct Border Zone in unloading group.
Figure S9: Significant enriched Pathway terms (TOP30) of differentially expressed and new miRNA of Normal Zone in unloading group.

## Figures and Tables

**Table 1:** Unloading evolution of unloading group.

Monitoring program	Before operation	AMI model established	FW-II type axial flow pump started
Cardiac output (L/min)	3.11 ± 0.06	2.73 ± 0.18	3.07 ± 0.03
Pulmonary wedge pressure (mmHg)	8.1 ± 1.8	9.6 ± 1.7	8.7 ± 1.2

**Table 2:** Statistics of miRNA number.

	Known miRNA	New miRNA
miRNA precursor	106	1021
miRNA maturity	152	1582 (815 were conservative)
