# Supplementary material for: The miRNA Expression Profile in Acute Myocardial Infarct Using Sheep Model with Left Ventricular Assist Device Unloading
Source: Biomed Res Int. 2017 Sep 11;2017:4352450. doi: 10.1155/2017/4352450 (PMC5611885; doi:10.1155/2017/4352450)
Supplement: Supplementary file 1 — Table S1: Differentially expressed and new miRNA of Infarct Zone in unloading group. Table S2: Differentially expressed and new miRNA of Infarct Border Zone in unloading group. Table S3: Differentially expressed and new miRNA of Normal Zone in unloading group. Figure S1: Significant enriched GO terms (TOP30) of differentially expressed and new miRNA of Infarct Zone in unloading group. Figure S2: Significant enriched GO terms (TOP30) of differentially expressed and new miRNA of Infarct Border Zone in unloading group. Figure S3: Significant enriched GO terms (TOP30) of differentially expressed and new miRNA of Normal Zone in unloading group. Figure S4: GO-Standard of differentially expressed and new miRNA of Infarct Zone in unloading group. Figure S5: GO-Standard of differentially expressed and new miRNA of Infarct Border Zone in unloading group. Figure S6: GO-Standard of differentially expressed and new miRNA of Normal Zone in unloading group. Figure S7: Significant enriched Pathway terms (TOP30) of differentially expressed and new miRNA of Infarct Zone in unloading group. Figure S8: Significant enriched Pathway terms (TOP30) of differentially expressed and new miRNA of Infarct Border Zone in unloading group. Figure S9: Significant enriched Pathway terms (TOP30) of differentially expressed and new miRNA of Normal Zone in unloading group. [file 4352450.f1.pdf]

Supplementary Materials

Compared with control group，there are many differentially expressed and new miRNAs in Infarct Zone(Table S1)、Infarct Border Zone(Table S2) and Normal Zone(Table S3) of unloading group

Table S1 Differentially expressed and new miRNAs of Infarct Zone in unloading group

Id	logFC
10_4323_mature	-3.318276771
11_6422_mature	1.838474331
11_6422_star	2.806337227
12_7254_star	2.100446356
12_7549_star	2.019684673
13_8425_mature	-1.876134772
16_11896_mature	3.186070721
20_21364_mature	1.872637088
26_26642_mature	3.381504503
5_32732_mature	-1.662667431
5_33100_star	4.413855003
7_36221_mature	-7.191336573
7_36266_mature	1.814707021
X_41026_mature	-3.31598557
X_41135_mature	-2.636926747
X_41195_mature	-1.876127706

Table S2 Differentially expressed and new miRNA of Infarct Border Zone in unloading group

id	logFC
1_1501_star	2.247490622
1_1873_star	2.408323781
1_2679_star	2.690348956
1_3067_star	2.406623804
1_3216_star	2.230224645

1_3395_star	2.632198767
11_4773_star	2.120149029
11_5692_star	2.282266467
11_5853_star	2.646351517
11_5943_star	2.694522286
11_5945_star	3.049634849
12_6792_star	2.625529614
12_7226_star	2.185613911
12_7398_star	2.29514019
13_8096_star	2.817301919
13_8171_star	2.096243804
13_8228_star	2.069934333
13_8234_star	1.985214306
13_8572_mature	2.121097715
13_8736_star	2.307002335
14_9009_mature	-3.465114862
14_9680_star	2.151854692
14_9865_star	5.529214731
15_11119_star	2.616751492
15_11175_star	2.323364551
18_13344_star	2.123631132
18_13617_star	2.260841606
18_14245_star	3.28681843
19_14404_star	2.507497488
19_15150_star	3.622965619
19_15152_star	3.623066256
2_15462_star	3.837592748
2_15652_star	5.450052284
2_15712_star	2.199893783
2_16066_star	2.164469143

2_16225_mature	-7.666578716
2_17871_star	3.21077677
2_18623_star	2.380842894
2_19086_star	2.355420384
2_19762_star	2.42725539
2_19905_star	2.308465564
20_20621_mature	4.393538246
24_24746_star	2.22573881
26_26761_star	2.057474248
3_27813_star	2.223425367
3_28053_star	3.538337342
3_28475_star	3.034792635
3_28602_star	2.791794419
3_28735_star	3.679918695
3_28766_star	2.214954801
4_31142_mature	-3.662436693
4_31559_star	2.833253455
4_32054_star	2.582128823
4_32219_star	2.737037282
5_32441_star	3.450561773
5_32704_star	2.304522076
5_33000_star	2.706739682
5_33003_star	2.266523541
5_33363_mature	-7.139913413
5_33569_star	1.993268091
5_34139_star	2.690346607
5_34196_mature	-4.265703473
6_34620_star	2.907196833
6_35042_star	2.942385986
6_35054_star	2.630004089

6_35314_star	5.455341873
7_36026_star	2.185638622
7_36027_star	3.24188986
7_36221_mature	-4.634397387
7_36419_star	2.180665585
7_36865_star	2.027645452
7_37134_star	2.630010306
9_38312_star	2.227575167
9_38727_mature	4.97437132
AMGL01127290.1_41372_star	2.427255493
JH922317.1_41340_star	2.67124661
X_40137_star	2.196495461
X_40140_star	2.386335223
X_40142_star	2.43329667
X_40162_star	5.098486172
X_40165_star	5.621506952
X_40171_star	4.175821866
X_40173_mature	-5.321591378
X_40187_star	4.608896062
X_40193_star	4.608873421
X_40298_star	2.903656789
X_40301_star	2.451231837
X_40328_star	2.161618288
X_40667_star	2.35442443
X_41135_star	2.676462448
X_41160_star	2.560147715
X_41190_star	2.054261163

Table S3 Differentially expressed and new miRNA of Normal Zone in unloading group

id	logFC
1_1501_star	-1.963477916
10_4201_mature	-3.819946541
11_4740_mature	4.061629883
12_7108_mature	-3.571110386
13_8171_star	-1.500450701
13_8425_mature	-1.952181004
14_9587_mature	-3.288351748
14_9865_mature	3.696247311
16_11698_mature	2.868926014
17_12519_mature	-1.987625589
17_13033_mature	-2.955403753
18_13344_mature	1.904288381
2_19009_mature	-2.567184136
20_21364_mature	1.86787302
23_24052_mature	4.939278124
24_25018_mature	2.2802155
3_27418_mature	2.541642281
3_27558_mature	-1.960961347
4_30931_star	2.59096899
5_32645_mature	3.122361383
5_32732_mature	-1.272936199
5_33100_star	2.886232129
5_34196_mature	-3.609074832
6_35160_mature	2.121966372
7_36221_mature	-3.826167882
7_36960_star	3.063259451
9_38773_mature	1.915847906
X_40165_mature	-2.585829219
X_40171_mature	-2.585833217

X_40177_mature	-2.894086655
X_40187_mature	-3.013400801
X_40193_mature	-2.894082104
X_41040_mature	2.133024067
X_41044_star	-1.975629946
X_41135_mature	-2.054366203
X_41195_mature	-1.952171723

The GO and KEGG Pathway annotations and enrichment were performed in differentially expressed and new miRNAs in Infarct Zone(Figure S1、S4、S7)、Infarct Border Zone(Figure S2、S5、S8) and Normal Zone(Figure S3、S6、S9) of unloading group.

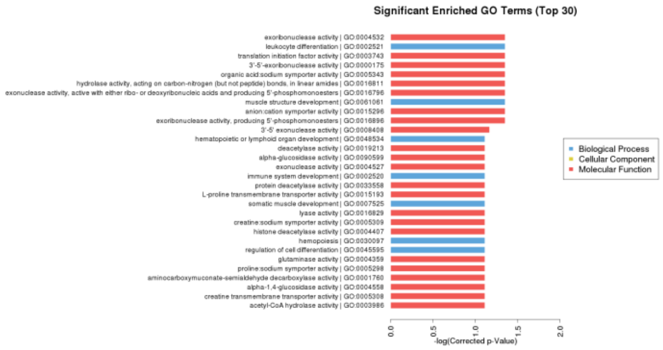

Figure S1 Significant enriched GO terms （ TOP30） of differentially expressed and new miRNA of Infarct Zone in unloading group

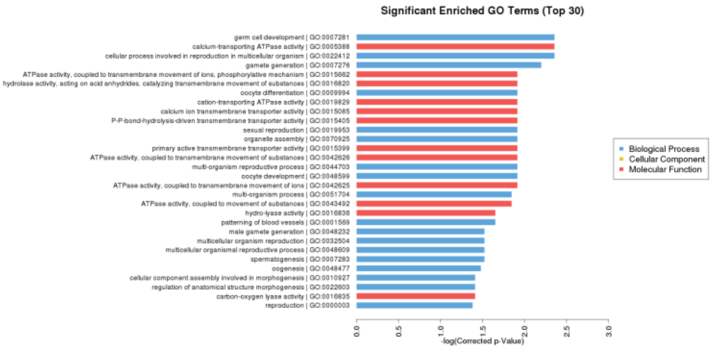

Figure S2 Significant enriched GO terms （ TOP30） of differentially expressed and new miRNA of Infarct Border Zone in unloading group

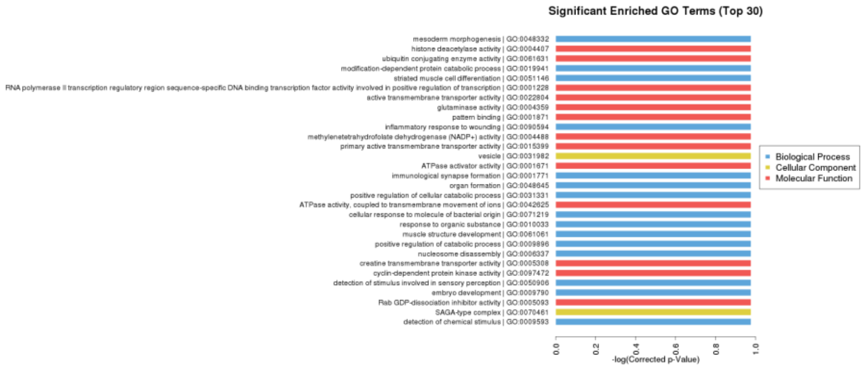

Figure S3 Significant enriched GO terms （ TOP30） of differentially expressed and new miRNA of Normal Zone in unloading group

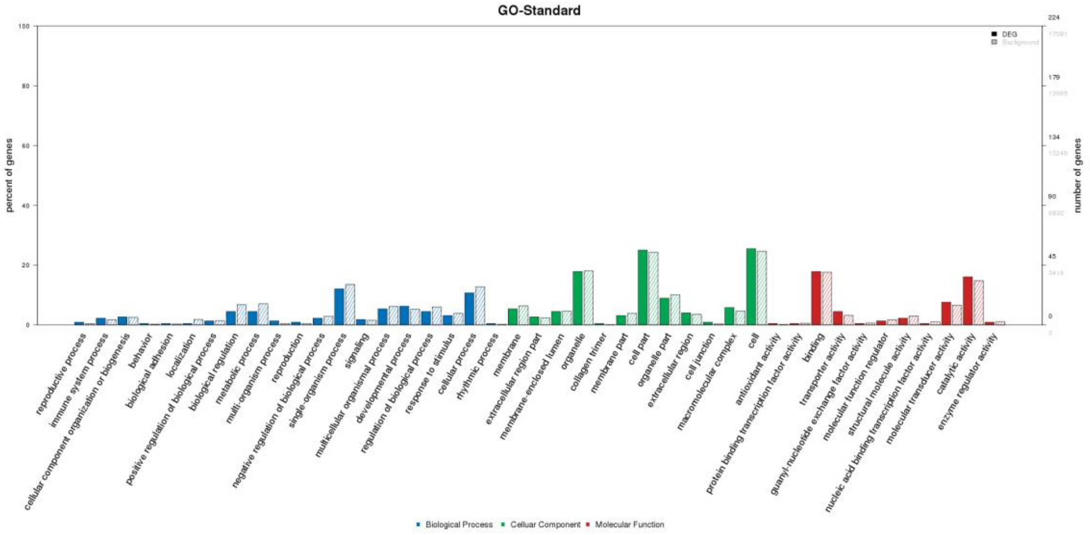

Figure S4 GO-Standard of differentially expressed and new miRNA of Infarct Zone in unloading group

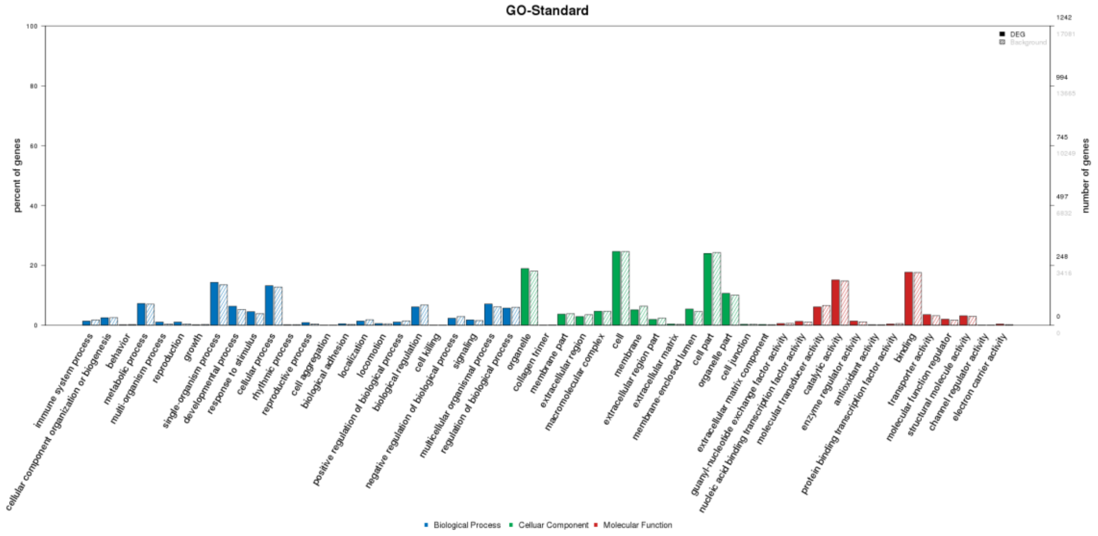

Figure S5 GO-Standard of differentially expressed and new miRNA of Infarct Border Zone in unloading group

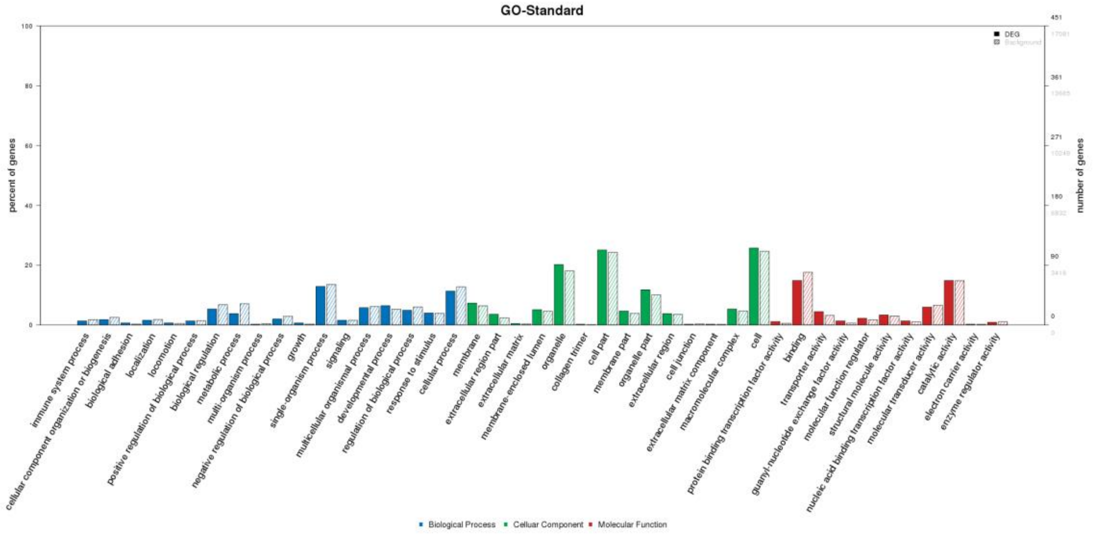

Figure S6 GO-Standard of differentially expressed and new miRNA of Normal Zone in unloading group

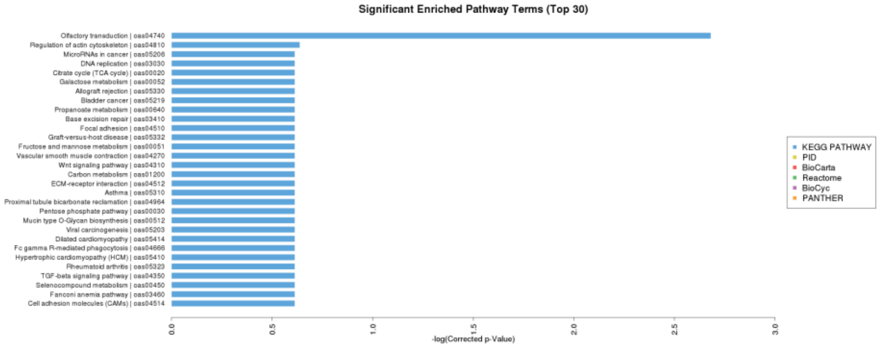

Figure S7 Significant enriched Pathway terms（ TOP30） of differentially expressed and new miRNA of Infarct Zone in unloading group

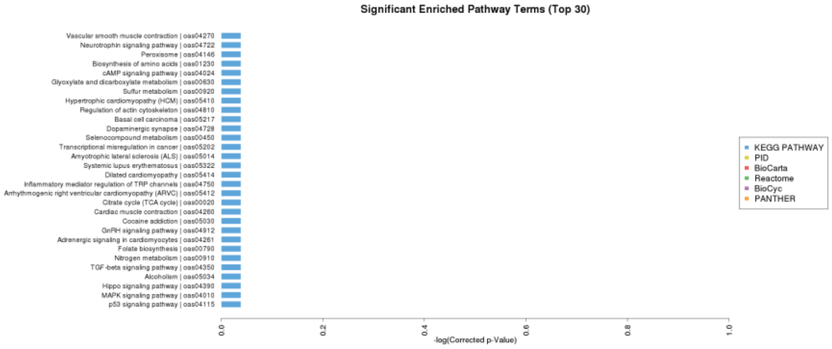

Figure S8 Significant enriched Pathway terms（ TOP30） of differentially expressed and new miRNA of Infarct Border Zone in unloading group

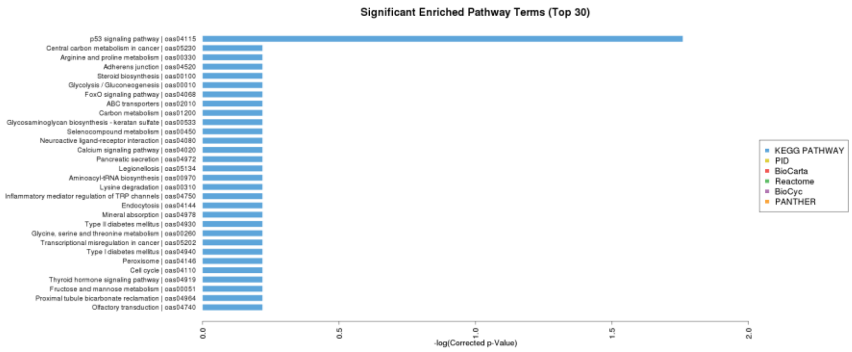

Figure S9 Significant enriched Pathway terms（ TOP30） of differentially expressed and new miRNA of Normal Zone in unloading group
